# Fluid-Structure Interaction Simulation of Prosthetic Aortic Valves: Comparison between Immersed Boundary and Arbitrary Lagrangian-Eulerian Techniques for the Mesh Representation

**DOI:** 10.1371/journal.pone.0154517

**Published:** 2016-04-29

**Authors:** Alessandra M. Bavo, Giorgia Rocatello, Francesco Iannaccone, Joris Degroote, Jan Vierendeels, Patrick Segers

**Affiliations:** 1 IBiTech-bioMMeda, ELIS department, iMinds Medical IT, Ghent University, Ghent, Belgium; 2 FEops bvba, Ghent, Belgium; 3 Department of Flow, Heat and Combustion Mechanics, Ghent University, Ghent, Belgium; University at Buffalo, SUNY, UNITED STATES

## Abstract

In recent years the role of FSI (fluid-structure interaction) simulations in the analysis of the fluid-mechanics of heart valves is becoming more and more important, being able to capture the interaction between the blood and both the surrounding biological tissues and the valve itself. When setting up an FSI simulation, several choices have to be made to select the most suitable approach for the case of interest: in particular, to simulate flexible leaflet cardiac valves, the type of discretization of the fluid domain is crucial, which can be described with an ALE (Arbitrary Lagrangian-Eulerian) or an Eulerian formulation. The majority of the reported 3D heart valve FSI simulations are performed with the Eulerian formulation, allowing for large deformations of the domains without compromising the quality of the fluid grid. Nevertheless, it is known that the ALE-FSI approach guarantees more accurate results at the interface between the solid and the fluid. The goal of this paper is to describe the same aortic valve model in the two cases, comparing the performances of an ALE-based FSI solution and an Eulerian-based FSI approach. After a first simplified 2D case, the aortic geometry was considered in a full 3D set-up. The model was kept as similar as possible in the two settings, to better compare the simulations’ outcomes. Although for the 2D case the differences were unsubstantial, in our experience the performance of a full 3D ALE-FSI simulation was significantly limited by the technical problems and requirements inherent to the ALE formulation, mainly related to the mesh motion and deformation of the fluid domain. As a secondary outcome of this work, it is important to point out that the choice of the solver also influenced the reliability of the final results.

## Introduction

With more than 25% of the elderly population (>65y) suffering of heart valve diseases in the US alone [1, 2], there is a great interest in the investigation of the valvular structures and their fluid mechanics. The aortic valve (AV) is one of the four passive heart valves, located between the left ventricle (LV) and the aortic root (AR), and regulates the blood flow between the left heart and the systemic circulation. During diastole, the AV is closed to prevent the backflow of blood from the aorta to the LV, and it bears an elevated pressure drop (about 140 mmHg in physiological cases, even higher in hypertensive cases). This enhances the risks of pathologies, most of which might lead to the replacement of the native valvular structure with an artificial prosthetic device. Prosthetic heart valves have been used and studied since the 1950s, when they were firstly developed and implanted [3]. Despite the long clinical experience and knowledge of the devices, there are still several drawbacks that need to be investigated in order to improve the performances and the outcomes of the surgical implantation. The more recent biological prosthetic valvular devices, manufactured with biological tissues (bovine pericardium or porcine valvular tissue), have been developed to guarantee a greater biocompatibility and a reduced interference with the haemodynamics of the AR when compared to the mechanical prosthetic valves. Nevertheless, they show durability issues as the tissue deteriorates with time, losing its mechanical properties. To understand the causes of the failure and provide relevant improvements to the design of these devices, computational modeling has been used as a convenient tool to simulate the operating conditions and to analyze both valve mechanics and haemodynamics in the region of interest. In this context, fluid-structure interaction simulation allows to investigate the mutual interplay of the soft tissues and the blood flow [4–13].

When setting up an FSI simulation, several choices have to be made to select the most suitable approach. To simulate flexible leaflets cardiac valves, a key parameter is the type of discretization of the fluid domain, which can be described with an ALE approach or an Eulerian formulation. In the Eulerian approach, introduced by Peskin in 1972 [4] for heart valve simulations and in the techniques based on the Immersed Boundary (IB) technique, the fluid domain is discretized with a fixed grid where the Navier-Stokes (NS) equations are written in the Eulerian formulation, while the structure is modeled with a Lagrangian mesh, free to move on top of the fluid domain. Following this approach other formulations have been proposed to improve the performances of this technique [3, 11–13]. For simplicity, in the following we will refer to these techniques with the general name of IB methods, being all based on the same underlying principles. On the contrary, when the ALE strategy is used, the fluid grid can deform according to the motion and deformations of the solid structure.

Several advantages can be counted using the IB over the ALE method: only the structural grid deforms, it has lower computational cost, and there are no issues related to a highly deformed fluid grid, making the technique more suitable for problems where the structural domain undergoes large displacements, as in case of heart valve dynamics studies [5–11, 13,14]. Furthermore, a very refined fluid mesh is required in the area where the movement is expected. On the other hand, this technique results in a less accurate description of the fluid-structure interface, where quantities such as the wall shear stress and pressure on the leaflets can be computed as potential pathological indicators [6, 9, 15]. These considerations suggest that theoretically the ALE technique would be preferable to perform heart valve simulations as the interface is sharply defined, and the variables are calculated directly on the surface and not obtained from the interpolation, as in the IB methods. An alternative would be to discretize the fluid grid with an Eulerian approach, while allowing a local mesh refinement in the proximity of the moving walls, in order to improve the quality of the definition of the surface in the fluid domain and overcome some of the major limitations of the IB formulation [6].

While the ALE approach has been used for vascular simulations [16], only few and simplified studies are reported in literature on valvular simulations. A 2D model of the AV with flexible leaflets based on the ALE formulation can be found in Chandra et al., 2012 [15], where the cycle is limited to the systolic phase and no contact of the leaflets is considered. Annerel et al., 2012 [17] performed a 3D FSI simulation of a mechanical prosthetic valve modeling the leaflets of the valve as rigid bodies. As such, their motion was calculated by means of angular displacement, with no need to solve the complete structural problem. To the best of our knowledge, no reports are available on full ALE-based FSI simulations of a 3D aortic valve with flexible leaflets. This simulation is commonly performed with IB-based methods, allowing for a faster solution, with no need of remeshing and managing the significant deformation of the fluid grid [5, 7–10, 13, 14, 18].

The goal of this work is to implement a model of a biological prosthetic aortic valve with flexible leaflets and perform comparable simulations with an ALE-based technique available within our group and the IB techniques as present in the commercial solver Abaqus/CEL, release 12.0. As a preliminary step, a 2D simplified model was generated. The model was then extended to a full 3D geometry, to perform the complete simulation. The paper is further organized as follows: in the methods section both the 2D and 3D models are described, highlighting the differences that were introduced between the IB and ALE formulation when necessary. The most significant results are then listed, focusing on the comparison between the two approaches. In the discussion and conclusion sections the comparison of the techniques is analysed, and the major limitations of the two approaches are discussed.

## Materials and Methods

### Arbitrary Lagrangian-Eulerian and Immersed Boundary formulation

**Fig 1:**
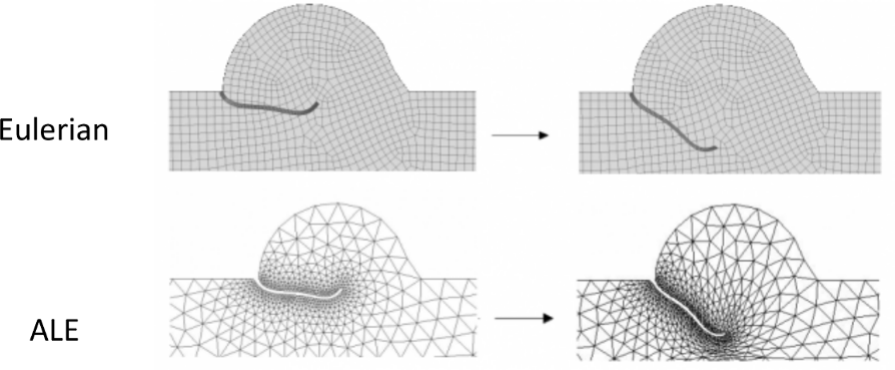
Grid formulation for the Eulerian (upper panel) and the ALE (lower panel) methods, in two consequent time-steps (from left to right) for the 2D aortic valve with flexible leaflets.

In both the ALE and IB methods the structural equations are normally solved in the Lagrangian formulation, while the type of discretization of the fluid domain is the most significant difference between the two approaches under investigation as depicted in Fig 1. From one time-step to the following (Fig 1, from left to right), the Eulerian mesh is fixed (Fig 1, upper panel) and does not deform when the structure (black) moves on top of it. The presence of the solid bodies immersed in the fixed grid is taken into account by the introduction of an external body force term in the NS equations in the fluid cells where the structure is located. By doing this, the effect of the structure is transferred to the underlying fluid with interpolating functions and no real interface exists in the fluid domain. On the other side, in the ALE framework (Fig 1, lower panel) the fluid mesh deforms following the movement/deformation of the solid body. The imposition of a no-slip condition at the boundary guarantees the equivalence of the fluid and solid grid velocity at the interface. Within each iteration, the grid displacement is extended from the solid boundary to the entire fluid domain with extension functions or by solving a system of equations resulting from e.g. a spring model or a pseudo-elasticity model [19]. In case of large deformations or displacements of the structure, the remeshing of the fluid domain is necessary to avoid highly distorted or inverted elements.

Due to the type of the discretization, in the IB model the interface between the two domains is not sharply defined. It exists only on the side of the solid domain, while in the fluid it is initially identified with the definition of the volume of fluid (VOF) function, i.e. the fraction of fluid which is allowed in each Eulerian cell due to the eventual presence of an immersed solid structure, and it is kept throughout the simulation by the enforcement of contact constraints and no-slip conditions. In the zone where the structure is located, the fluid cells contain a percentage of void resulting in a partially or completely empty cell, to account for the presence of the structure at the same location. Conversely, in the ALE case, the surface is geometrically defined in the fluid domain and it is allowed to deform along with the structural mesh. The interaction between the two domains is obtained by the imposition of the kinematic (Eq 1) and dynamic (Eq 2) constraints at the interface.

$$\vec{v_{f}}=\frac{d\vec{u_{s}}}{dt}$$ (1)

$$\sigma_{f}\cdot \vec{n_{f}}=-\sigma_{s}\cdot \vec{n_{s}}$$ (2)

where $\vec{v_{f}}$ and $\frac{d\vec{u_{s}}}{dt}$ are the grid velocities at the interface *Γ*_*i*_, *σ*_*f*,*s*_ are the traction forces at the interface and $\vec{n_{f,s}}$ are the normals pointing outwards of the fluid and structural domain, respectively.

In the current study we compared the performances of the two techniques in a 2D and 3D example case. The model was kept as similar as possible in the two settings for comparison purposes within the limitations and the requirements of the specific solver and technique. The model-specific differences are highlighted in the following sections when necessary.

### FSI Solvers

In this work, all the IB-based simulations were performed within the Abaqus/CEL (Coupled Eulerian-Lagrangian) environment, an extension of the module Abaqus/Explicit version 12.0 (Dassault Systèmes, Providence, RI, USA). This module does not couple multiple software products, but solves the interaction simultaneously within Abaqus [20], imposing the interaction between the two domains with contact constraints.

The ALE simulations were performed with a strongly coupled and partitioned solver, which makes use of an in-house written coupling algorithm (using the Interface Quasi-Newton technique with a Least-Squares model, as implemented in Tango [21]) to couple any two black box solvers. In particular, Fluent (Ansys, release 15.0) and Abaqus/Standard version 12.0 (Dassault Systèmes, Providence, RI, USA) were used in this work. The convergence criterion for the coupling algorithm in the case of interest was the reduction of the norm of the change in interface displacement with a factor 10000 relative to the value obtained in the first coupling iteration. The performance of this FSI framework has been tested and verified in numerous works with different applications, including the biomedical field [16, 21, 22]. With regard to the deforming domain of the ALE-FSI simulation, the mesh deformation was managed by the solver Fluent. Separate tests were performed to identify the most adequate set of parameters for the smoothing algorithms necessary to preserve a good quality of the fluid mesh.

### 2D and 3D models

**Fig 2:**
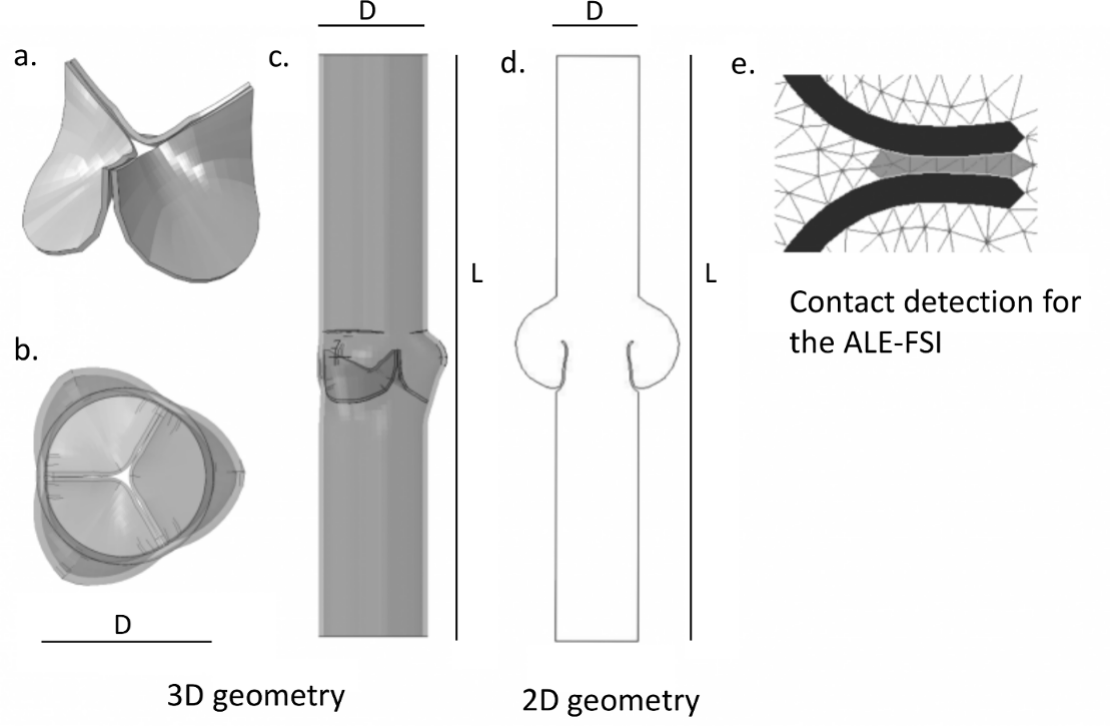
**(a)** Geometry of the bioprosthetic valve, (**b,c):** Three-dimensional geometries of the entire domain, (**d)** Two-dimensional domain. (**e)** Contact detection function for the ALE-FSI simulation. In black the leaflets, in white the fluid domain, and in grey the cells detected for contact.

A Carpentier-Edwards PERIMOUNT Aortic Heart Valve (Edwards, Lifesciences LLC, Irvine, CA) was scanned with a μCT scan Triumph-II imaging system (TriFoil Imaging, Chatsworth, CA). The obtained images were segmented with the commercial software Mimics (Materialise NV, Leuven, Belgium) to obtain the desired 3D volume and geometry (Fig 2A). To obtain the entire domain, the valve was placed into a straight and rigid tube (length L = 130 mm) with three hemispherical enlargements (Fig 2B and 2C diameter D = 20 mm), to represent the sinuses of Valsalva [23]. The 2D model was obtained as an idealized section of this geometry, and included two symmetric leaflets placed in a straight rigid tube with two enlargements (Fig 2D). The dimensions were consistent in the IB and ALE models, and were chosen according to literature data.

The structural mesh was similar in both models and consisted of quadrilateral or hexahedral solid elements, for 2D and 3D simulations respectively. Due to the requirements imposed for the generation of an adequate fluid mesh in the IB-FSI case, it was necessary to reduce the number of the solid elements in the IB-FSI case to consequently increase the element size. More details are provided in the following sections. The fluid mesh of the ALE simulation consisted of triangular (2D) or tetrahedral (3D) elements with a higher cell density in the vicinity of the valve. Due to the ALE formulation, it was possible to obtain a non-uniformly spaced fluid grid, which reduced the overall number of elements for this mesh. The fluid mesh of the IB-FSI was made of quadrilateral (2D) or hexahedral (3D) elements. To have a satisfactory definition of the initial VOF in the IB method, a homogeneously refined fluid mesh had to be generated. This resulted in a much more refined mesh for the IB-FSI than for the ALE-FSI. To limit the global number of Eulerian elements in the IB-FSI simulation, the fluid domain was shortened to 30 mm. The final mesh used for the IB-FSI simulation resulted in a denser and larger mesh, if compared to the mesh used in the ALE-FSI case, as reported in Table 1. In Fig 1 (upper panel) and 1 (lower panel), the 2D fluid meshes for ALE-FSI and IB-FSI are reported, respectively. Due to visualization difficulties, the 3D fluid meshes are not reported here. Adequate dimensions of the meshes were chosen for the different set-ups. The number of elements is listed in Table 1. For the ALE simulations, the initial dimension of the fluid meshes is reported, as the remeshing of the domain was enabled and the number of cells thus varied during the simulation.

**Table 1:** Number of the mesh elements in the different set-ups.

	2D		3D	
	ALE-FSI	IB-FSI	ALE-FSI	IB-FSI
SOLID	500	300	5200	1250
FLUID	2400	3500	150000	1150000

### Material properties and boundary conditions

The leaflets tissue was modelled as linear and elastic (Young modulus 1 MPa, Poisson ratio 0.45) [5, 8, 10], while the elasticity of the aorta was neglected and the arterial walls were assumed to be rigid [5, 17]. For the comparison purpose of this work we considered these simplifications to be justified, while for a more refined model the elasticity of the wall has to be included and more realistic material models of the soft tissues are necessary [9, 13, 24, 25, 26]. The blood was modelled as a Newtonian fluid (density 1060 kg/m^3^, viscosity 0.003 Pa∙s). In the IB-based simulation, a compressibility factor was added to the fluid to enhance the convergence of the solution [8,9,14]. This coefficient was set by imposing a speed of sound in the fluid c_f_ of 157 m/s in the 2D case, and of 15.7m/s in the 3D case (with the effective value being in the order of 1570 m/s). A physiological transvalvular pressure difference curve was applied at the ventricular side of the domain, while the aortic outlet was kept at a reference pressure. Before the loading cycle, the pressure was gradually increased until the pressure of the cycle was reached to provide a good initial condition for the simulation [27] and to reduce the influence of the compressibility of the flow [9]. A no-slip condition was imposed on the walls, while the fluid-structure interaction condition was enforced in the leaflets region.

In the ALE-FSI, a fixed time-step size was chosen. To capture the dynamics of the valve motion and to guarantee the contact detection in the structural solver a time step of 0.1 ms was selected. The time integration scheme used was a first order, implicit method. In the IB-FSI, an automatic and adaptive time-step was selected, and an explicit integration scheme was used. An initial time-step size of 0.1 ms was chosen, coherently with the ALE-FSI. The time-step size was then automatically calculated and updated throughout the simulation by the solver.

### Contact

The management of the contact in the two models was substantially different. The solid-solid contact in the IB-FSI was directly managed by the solver Abaqus via a default contact penalty method [20]. The contact in the ALE-FSI was not available automatically: an *ad hoc* function was introduced to detect the areas experiencing contact, to hamper the motion of the valve and to preserve a one-layer-cell gap between the leaflets. During contact, in fact, the leaflets of the valve should close completely, and, due to the ALE formulation, this would result in the generation of highly distorted elements and, ultimately, the splitting of the fluid domain and failure of the simulation. To avoid this phenomenon, it was necessary to preserve a layer of fluid cells in the contact area of the leaflets. The kinematic constraint to hamper the motion of the valve was imposed in the structural solver with the default contact algorithm available in Abaqus/Standard. The properties of the contact were consistent with the properties of the contact definition of the IB-FSI. A minimum threshold distance between the leaflets was imposed, to preserve a gap during the diastole. However, on the fluid side, the presence of the gap introduced an unwanted and artificial leakage of the valve when it was in the closed configuration. As we previously demonstrated, valve leakage can be reduced by modifying the permeability of the cells located in the area during the coaptation time [28]. The hydraulic resistance was increased in these cells, to reduce the overall backflow during diastole. This could be obtained by implementing a specific external function in the fluid solver, which detected the cells of the gap and changed their properties. This function was activated only during the closed phase of the valve, when the leaflets came closer than a predefined threshold. This technique does not intend to mimic any physiological phenomenon and the permeability coefficients were chosen as a trade-off between the reduction of the backflow and the stability of the solution. The 2D visualization of the contact function is depicted in Fig 2E. The cells marked in grey were selected for contact and a kinematic constraint was imposed to avoid the domain splitting. The permeability of these cells was modified (only during the contact phase) to artificially increase their hydraulic resistance to the flow.

## Results

In this section, we report the comparison between the most significant results of the two techniques in the 2D and 3D case.

### 2D simulations

**Fig 3:**
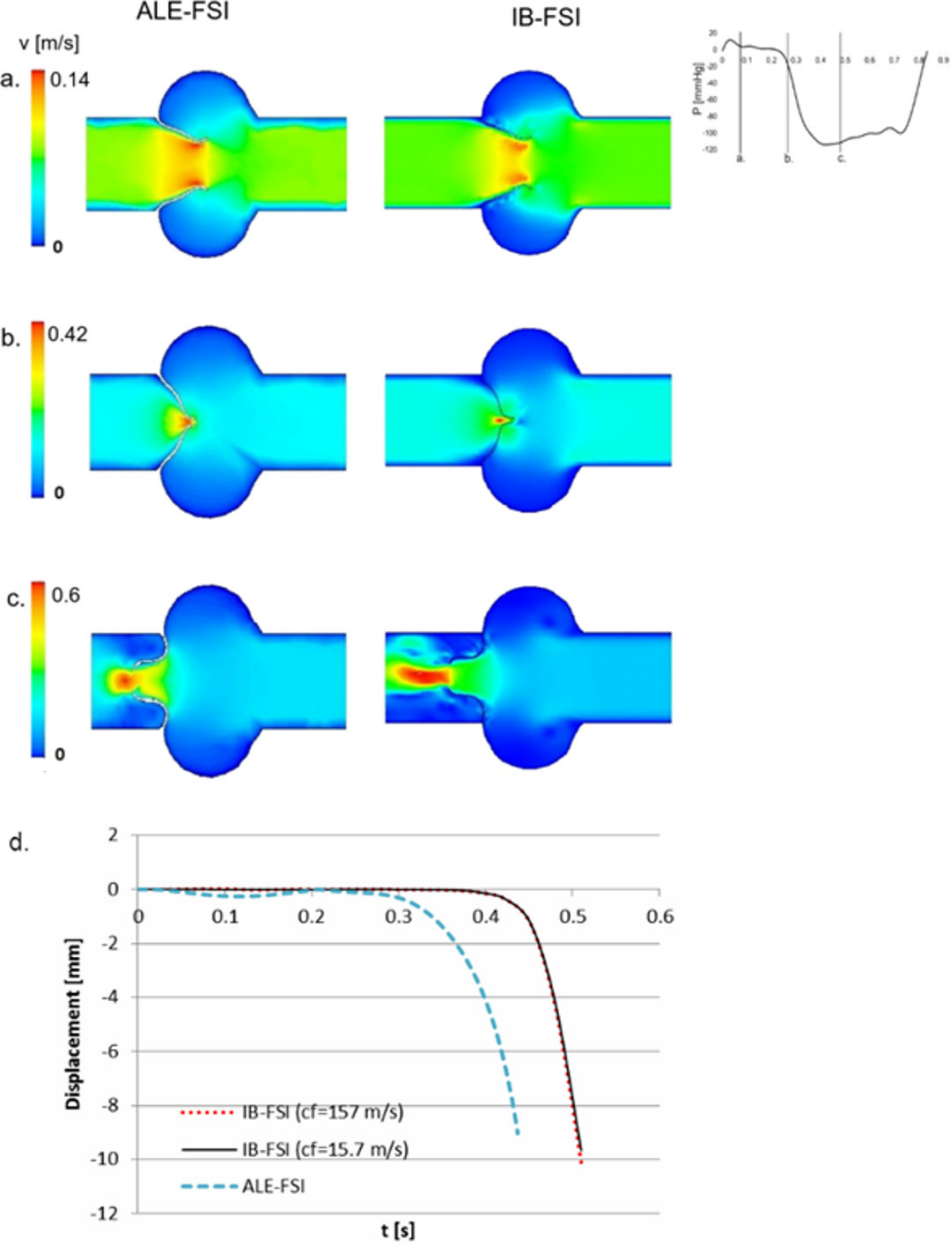
Comparison between the ALE and the IB results at (a) late systole, (b) closure phase, (c) early diastole, (d) comparison of the displacement of the nodulus of Arantio in the ALE-FSI case and in two IB-FSI simulations, obtained with cf = 157m/s (red) and cf = 15.7m/s (black).

The overall 2D flow field and valve kinematics were comparable between the two techniques. The open valve was assumed to be the initial configuration. Being already open, the valve did not offer any resistance to the fluid during the opening phase, therefore we report the results of the closing phase only, until early diastole. The flow decelerated when the pressure drop across the valve reversed, and the valve started to close (Fig 3A). After about 0.4s the valve was in the closed position and a small central leakage was detected, especially in the ALE-FSI case (Fig 3B). The IB-FSI simulation showed a small time delay in the kinematics of the valve: in this case, in fact, the leaflets reached the closed position with a delay of maximum 50 ms in comparison to the ALE-FSI. As visible from Fig 3C, the 2D case was not suitable to simulate the diastolic configuration of the valve. In diastole, the pressure drop across the valve is significant, and the thin 2D structure was not able to keep the closed configuration, even in presence of the contact condition. For this reason, the 2D simulation was limited to the closing phase of the valve, and only one cardiac systole was simulated. The IB-FSI simulation was performed with a speed of sound in the blood c_f_ = 157.0 m/s, coherent with previously reported values [9, 14]. This value had to be reduced to c_f_ = 15.7 m/s for the 3D set-up. To verify the influence of the increased compressibility assumption, the comparison between the two cases (c_f_ of 157 m/s versus 15.7 m/s) was performed in the 2D model. In Fig 3D the displacement of the nodulus of Arantio of the valve is reported, for the ALE-FSI (in blue) and for the IB-FSI (red and black for the simulations with c_f_ = 157m/s and c_f_ = 15.7m/s, respectively). The delay of both the IB-FSI simulations with respect to the ALE-FSI is visible, while no difference in the timing is detected for the different values of the speed of sound.

### 3D simulations

**Fig 4:**
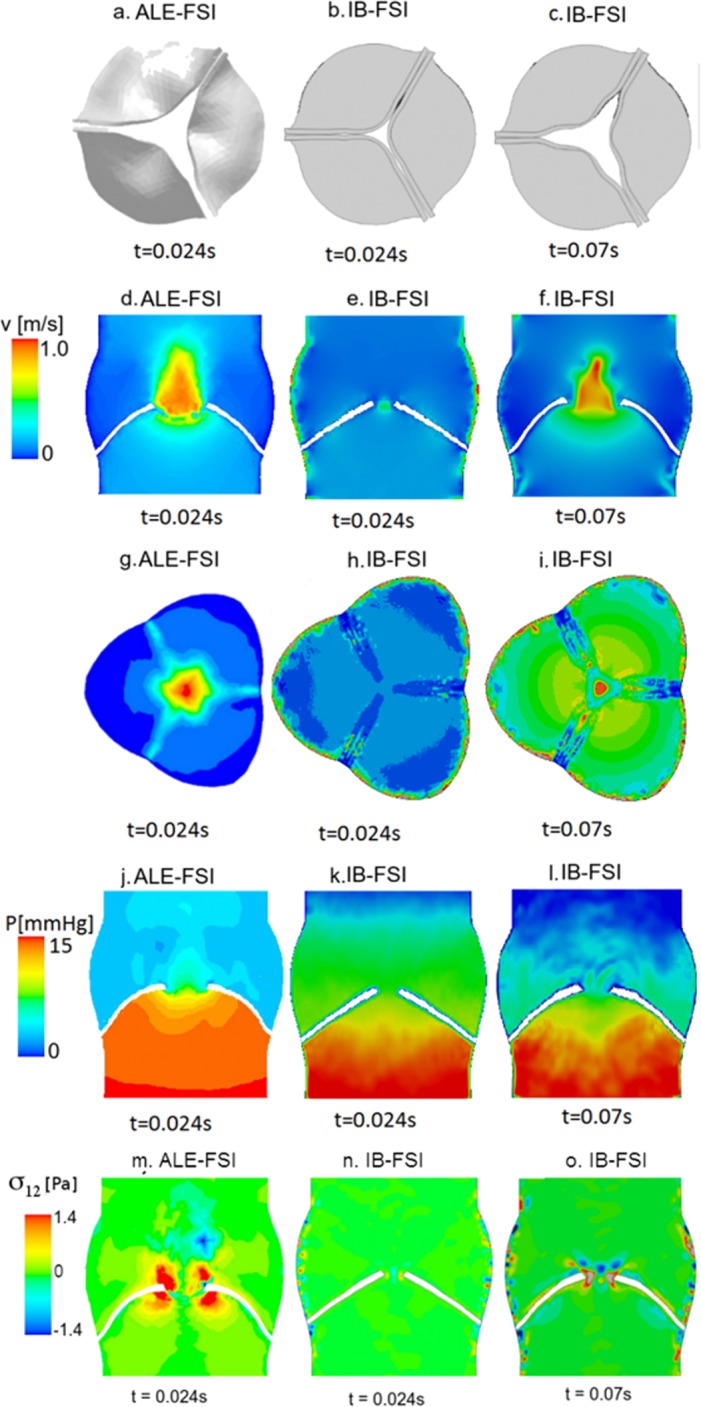
(a) Maximum open configuration achieved with the ALE technique at t = 0.024s. (b) IB-FSI valve at t = 0.024s. (c) IB-FSI valve at t = 0.07s. (d-f) Velocity profile on a longitudinal section. (g-i) Velocity profile on the cross section, (j-l) pressure distribution on a section. (m-o) Shear stress *σ*_12_ on a section.

The initial configuration for the 3D model was assumed to be the closed position, being the prosthetic valve in this configuration during the scanning. The major issue encountered in this comparative work was related to the difficulties in simulating the opening of the 3D valve in the ALE-FSI case. The fluid grid underwent severe deformation in a limited amount of time. Significant remeshing and smoothing algorithms to preserve a good quality grid for the fluid domain was not sufficient to ensure the convergence of the problem. The ALE-FSI failed at the early opening phase (t = 0.024 s) because of the generation of inverted volume cells. In Fig 4A, the maximum open configuration achievable with the ALE-FSI is reported. On the contrary, the valve in the IB-FSI reached the fully open configuration. Due to the limited availability of the ALE-FSI results, the comparison between the two methods was reduced to the early systolic phase. In Fig 4 the results are shown for time t = 0.024s (Panels d, e, g, h, j, k, m and n), and for time t = 0.07s (panels c, f, i, l and o), time point in which the IB valve was in the same configuration as the ALE-based valve. At t = 0.024s the ALE-based simulation (Fig 4D) showed higher velocities as compared to the Fig 4C (same time-point in the IB-FSI). Also, the deformation of the valve leaflets was different: the valve in Fig 4A and 4D has the classic bulged shape of the leaflets protruding into the aortic region during the opening phase, while in Fig 4B and 4E the valve is almost in the initial position. To have a similar open configuration in the IB-FSI in terms of valve position and velocity field it was necessary to wait until t = 0.07s, thus the delay that the IB-FSI has in comparison to the ALE-FSI was detected also in the 3D set-up. In Fig 4E a layer of higher velocities is detected in the proximity of the walls. This phenomenon was detected in all the time-points of the IB-ALE simulations and is related to a visualization issue of the software (it should not have any influence on the results) [20]. At this stage of the cardiac cycle, no blood recirculation in the Valsalva sinuses area was detected, as the leaflets were still in the opening phase. The recirculation zone formation was present in a later phase of the systole, when the AV started the closure phase. As this effect was only visible in the IB-FSI, the results are not reported here. The considerations about the pressure distribution of Fig 4 (panels from j to l) are similar to those for the velocities in Fig 4, panels d-f. At time t = 0.024s (Fig 4D, 4G and 4J) the difference in the pressure distribution in the two simulation types is evident. It is also possible to notice the presence of a thin layer of low pressure in the neighbourhood of the walls (leaflets and aortic root) in the IB-FSI, which is not visible in the ALE-FSI result. Furthermore, some variations of the pressure are visible in the area downstream the valve in the IB-FSI. In Fig 4 panels m-o, the comparison of the shear stress component *σ*_12_ is proposed for the two models. In the zone between the leaflets, an area with higher *σ*_12_ values was detected in the two set-ups, related to the higher velocity of the fluid. On the aortic side of the leaflets a small area with higher *σ*_12_ values was detected, due to the velocity of the opening leaflets. The presence of localized values of *σ*_12_ at the aortic walls in the IB-FSI is related to the presence of visualization artefacts [19].

**Fig 5:**
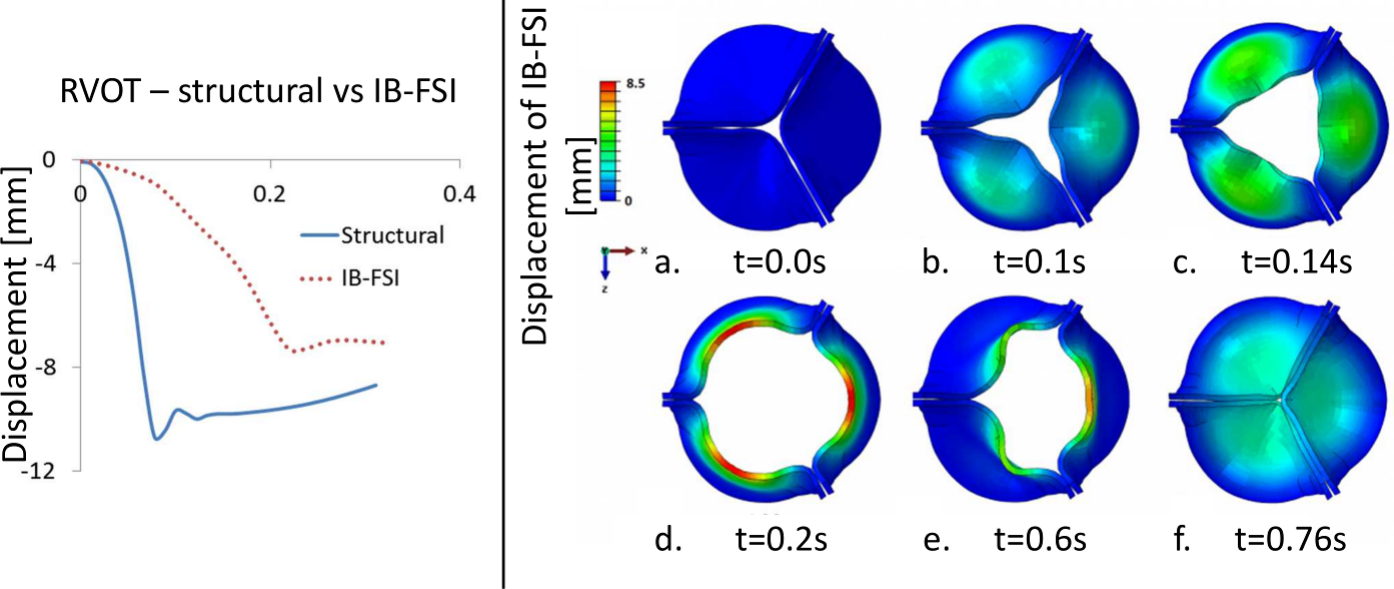
Left panel: displacement of the nodulus of Arantio, comparison between an IB-FSI and a structural simulation. Right panel: kinematic of the leaflets in significant time-points of the heart cycle until diastole (IB-FSI). The colour scale indicates the magnitude of the displacement calculated from the initial configuration.

Comparing the rapid valve opening time (RVOT) of the IB-FSI (200 ms) with literature data (reported range 45 to 65 ms, both from structural [29–31] and from FSI [8–10] simulations), the time delay previously described in the 2D comparison was even more pronounced in the 3D case. To verify the source of delay, a pure structural simulation was performed on the same geometry. The model was consistent with the IB-FSI simulations in terms of geometry, mesh, material and element type. In the structural case the loading pressure curves were directly applied on the leaflets surface. In Fig 5 (left panel), the displacement of the Nodulus of Arantio is reported for the IB-FSI and the structural simulation. The obtained RVOT in this case was about 80ms, significantly closer to the expected value but remaining outside the physiological range for this parameter.

For the sake of completeness, in Fig 5 (right panel) we report the displacement of the valve during systole (up to the beginning of diastole) obtained with the IB-FSI [32]. The flow field results are omitted for conciseness reasons.

**Fig 6:**
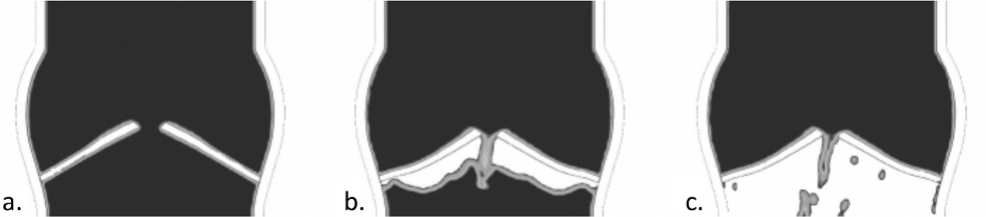
Loss of the definition of the VOF in the domain in case of high and reversed ΔP across the valve. In black the cells filled with fluid, in white the cells filled with void.

Focussing on the leaflet kinematics resulting from the IB-FSI simulations, the opening phase lasted from t = 0 s to t = 0.2 s (Fig 5A–5D), with the typical orifice shape that was detected in the AV opening. At t = 0.2s the valve was in its fully open configuration (Fig 5D). As shown in Fig 5E, when the ΔP across the valve reversed, the leaflets movement ceased to be symmetric (t = 0.6s). This was also verified by following the displacements of the central part of the aortic leaflet (nodulus of Arantio). At t = 0.76s the valve was in the fully closed position, with the contact enforced in the coaptation zone (Fig 5F). A residual central opening was visible. To investigate the unexpected asymmetric motion of the valve, a separate test on the diastolic phase was performed, where the valve was kept in the closed position and the pressure on the aortic side was gradually raised from the reference pressure to a value of 120 mmHg, while the ventricular pressure was kept at the reference pressure. By analysing the definition and the evolution of the VOF in this set-up, a loss of void (Eulerian cells in which no fluid is defined, in white in Fig 6) was detected on the ventricular side of the valve. In Fig 6, this phenomenon is illustrated in more details in Fig 6A the initial definition of the VOF is shown. By increasing the transvalvular pressure (Fig 6, panels b and c), the empty cells propagate in the ventricular portion of the fluid domain.

This effect was noticed in both the simulation of the diastole and in the complete cycle. In the latter, the loss of void was not symmetric, therefore causing the asymmetry of the leaflets kinematics shown in Fig 5. This phenomenon made the diastolic results unreliable, reason why the simulation was stopped at the beginning of the diastolic phase, and the simulation of one single cardiac systole was performed. This phenomenon had no physical meaning, it was a numerical issue related to the model discretization.

### Computing time

All the IB-FSI simulations were performed on a cluster (3.4 GHz and 5.6GB of RAM), in a parallel process on 8 CPUs. All the ALE-FSI were performed on a Dell PowerEdge R620 server (2x Intel Xeon E5-2680v2 CPUs at 2.8 GHz). There was a clear advantage in using the IB method in terms of computing time. Despite the use of a much larger computational grid for the Eulerian case (Table 1), the simulation resulted to be much faster than the ALE case, as there was no need of remeshing and no issues related to a highly deformed mesh. In addition, the in-house coupling code might require further optimization to deal with a complex scenario as the described problem. In Table 2 the required time is reported for each case.

**Table 2:** Computational time required by the two techniques. In the 3D case, for the ALE the time of the partial simulation is reported, while for the IB-FSI the time for the complete simulation is listed.

2D		3D	
ALE-FSI	IB-FSI	ALE-FSI	IB-FSI
48 h	1h	> 3 weeks (early opening)	30 h (entire simulation)

## Discussion

### 2D simulation results

In the 2D case, the comparison of the two models provided similar results, despite the presence of some time delay in the IB-FSI case. The kinematics of the valve was comparable to the literature data [15, 33, 34] and the discretization technique does not introduce significant differences between the ALE and the IB case. Also, the ALE fluid solver is capable to automatically manage the smoothing and the remeshing of the computational mesh. A simplified but similar comparison can be found in Dos Santos et al., 2008 [11], showing an analogous behaviour of a 2D flexible slab moving in a fluid domain with the ALE or the Eulerian approach. Even though the time required for the IB-FSI (1 hr) was lower than for the ALE-FSI (48 hrs), to our experience the latter was still considered to be in a reasonable range for an FSI simulation. Due to the simplified model and its 2D nature, it was not possible to simulate the diastolic phase: the two thin structures of the leaflets could not bear the high pressure difference imposed across the valve, and even with the imposition of the contact constraints they reversed into the ventricle. The same behaviour was detected in both the IB- and ALE-FSI simulations during diastole. To verify that this inability to simulate the diastolic phase was not related to the FSI technique as such or the way the contact between structures was defined, a pure structural 2D simulation was performed in Abaqus/Standard in which the geometry, element type, material properties and the contact properties were consistent with the FSI simulations. A transvalvular pressure difference was imposed between both sides of the leaflets. Two different cases were tested to check the potential impact of the definition of the contact properties: in the first case a gap between the leaflets was allowed (as done in the ALE-FSI), in the second case no gap was kept and the valve could completely close. In all the tested cases the structure reversed in the ventricular side of the tube during diastole. Therefore, we could conclude that the buckling of the structure was mainly related to the two dimensionality of the problems, and not on the fluid grid discretization or the contact function used. We hypothesize that this phenomenon was no shortcoming of the numerical codes, but rather due to the fact that a closed valve in the 2D configuration was physically not possible under the assumed boundary conditions and leaflet properties, which would lead to valve prolapse.

### 3D simulation results

Even though in theory the ALE-FSI would be preferable to obtain results that are precise and in which the surface of interest is sharply defined [6], in our experience this approach was significantly limited by the large deformations of the fluid grid, which were the cause of the failure of the simulation. The mesh motion algorithms tested were the spring-based model and the diffusion model. The required parameters for the two algorithms were chosen according to the characteristic dimensions of the fluid mesh. Several tests were performed to increase the mesh density and decrease the time-step size to avoid the excessive deformation of the mesh, leading to cells with negative volume. As the chosen grid was unstructured and the motion was complex and not known *a priori* but defined by the interaction of the two domains, it was not possible to calculate the displacement of the structure prior the calculation and adjust the time-step size and dimensions of the fluid grid accordingly [35]. Different initial configurations for the valve have been tested, starting from a slightly open position to a fully open position, but the appearance of negative volume cells arose any time the large displacement of the valve occurred. Besides these aspects, the required computational time for the (partial) ALE-FSI is considered to be excessive compared to the performances. We can therefore conclude that the IB approach is preferred over the ALE approach, at least with the tested fluid mesh motion algorithms in the case of large deformations of thin structures. Whether it is totally infeasible to perform ALE-FSI simulations of the 3D problem is difficult to state. What we do know is that, with the settings tested in this work, it is not practically possible to simulate the complete opening and closing of a flexible leaflet aortic valve. Alternative solutions to alleviate this problem can be, among others: (1) the use of alternative available smoothing algorithms, for example the solid elastic based smoothing algorithm, (2) the motion of the internal points of the fluid domain could be controlled by the user. However, there is no guarantee that these approaches will eliminate the problem of negative cell volumes.

### Solver choice

The choice of the solver has an important impact on the outcomes of the model. According to our experience, a solver as Abaqus/Explicit (release 12.0), primarily made to solve pure structural problems, it is not the most appropriate choice in case of IB-FSI simulations for heart valves. In particular, the definition of the VOF in the IB-FSI problem resulted to be the bottleneck of this type of simulation. To have an adequate definition of the VOF, the size of one fluid element had to be smaller than one third of the structural element [20]. Due to the high complexity of the motion of the structure, the refinement of the fluid mesh had to be done in the three dimensions and extended to the entire region where the valve is expected to be, which is why the Eulerian fluid mesh resulted to be one order of magnitude larger than the ALE-mesh (Table 1). By increasing the cells density of the Eulerian mesh, the results showed an improvement, but some of the issues remained, e.g. the loss of void when the pressure drop across the valve reversed. In our opinion, the loss of void could be referred to the VOF definition, which in our case seemed to be not yet satisfactory. This phenomenon had no physiological meaning, therefore the analysis of the results was limited to the opening phase of the valve. In the current study it was not possible to obtain an even more refined fluid grid: the element size would have been too small and the solver would have no longer been able to distinguish two neighbouring nodes. As the valve is a thin and highly flexible structure, shell elements might be a more suitable choice for the model and could remove some constraints on the requirements for the fluid mesh, allowing for larger fluid elements. A separate investigation showed that this type of elements is probably not optimized for fluid-structure contact yet in Abaqus/CEL version 12.0, and therefore the simulation degenerated with distorted elements [32]. The choice of using continuum elements was one of the limiting factors of this work: their small size influenced the dimensions of the fluid domain cells, leading to the above-mentioned problems related to the VOF. For the same reason, only one layer of cells was present in the thickness of the leaflets: thus a numerical stiffness was introduced in the leaflets, especially regarding the bending modes of the structure. The use of hourglass controls and mass scaling factors did not improve the overall performance of the simulation. The use of quadratic elements is currently not available in Abaqus/Explicit [20].

One of the possible alternatives to solve the problems related to the IB-FSI formulation and overcome the correct definition of the VOF would be to allow a partial adaptation of the fluid mesh in the vicinity of the moving walls [6, 10–12], to guarantee a finer mesh which can follow the movement of the structures and allow a coarser mesh in the remaining of the flow domain.

### Compressibility factor

In the IB-FSI, the equations that are solved for the Eulerian elements are initially written for highly deformable and plastic materials: the fluid-structure interaction is therefore an extreme case in this category, where the plastic material is a fluid. This introduces convergence issues in the IB-FSI model, which were solved with the introduction of a small compressibility of the blood commonly adopted [8–10] to enhance the convergence of the FSI simulations. In physiology, the speed of sound in the blood c_f_ is equal to 1570 m/s, in our 2D case the amount of compressibility introduced resulted in a c_f_ = 157 m/s, which was comparable to the values reported in literature [8, 9, 14]. This was not the case for the 3D setting, where the chosen value for the compressibility of the blood is higher than the values reported in literature (c_f_ = 15.7m/s vs c_f_ = 157 m/s). Due to the complexity of the 3D case, the choice of the element type and the requirements for the fluid mesh, the choice of higher values of c_f_ would lead to instabilities of the simulation. Since reducing of the speed of sound to such a low value could affect the reliability of the results, additional investigations were conducted to verify the validity of our choices. In a set of separate preliminary tests on the 2D set-up, the displacement of the leaflet was monitored to investigate the influence of the augmented compressibility, by performing the same FSI simulation with the two chosen different values for c_f_. The results were comparable, both in terms of displacement and opening time, suggesting the independence of the results from the chosen compressibility factor [32], as shown in Fig 3D. The Mach numbers obtained in all the presented simulations were within the range of 0.006 to 0.06, falling in the range of incompressible flow. By comparing the obtained Mach number with the results available (where the compressibility was included), the results showed a good agreement [9, 15, 36, 37]. To further verify the influence of the compressibility factor in the case of interest, a separate test of numerical experiments is reported in S1 Appendix. The mass flow balance of the simulations was verified, the imbalance between inlet and outlet was below 1% of the outlet flow throughout the duration of the simulation for the worst case of c_f_ = 15.7m/s.

### Time delay

In both the 2D and 3D simulations, the IB-FSI showed a time delay in the valve kinematics, when compared to the ALE-FSI in the 2D case, or to literature data in the 3D set-up. In the 3D case this delay was significant: the calculated RVOT was about 200ms, against an average of 55 ms of RVOT from previous FSI studies in the literature [8, 9, 29–31]. *In vitro* studies conducted on a similar type of valve on a pulsatile artificial circulation system report a RVOT for a Carpentier Edwards Perimount Magna valve of 48±10ms [38, 39]. The comparison of the opening time of the aortic valve with *in vivo* reports is more challenging, as the boundary and working conditions are not easily comparable. Typically, the ultrasound recordings are performed in the case of (stenotic) pathologies. Nevertheless, values of 57.5±11.1 ms [9, 40] are reported. All available data thus seem to indicate that the opening time of about 200 ms, resulting for our 3D IB-FSI simulations, is an overestimation, although it is to be stressed that the only conclusive experiment would be a one-on-one comparison between experimental observations and numerical simulations using an identical set-up (valve and cardio-vascular geometry, liquid properties, …) and boundary conditions.

Several tests were performed to verify the origin of the delay: despite the artificial compressibility factor was shown to be not relevant for the IB, it might still have a residual effect when dealing with complex highly dynamic events such as the valve motion [32]. The choice of the elements type also played an important role: due to the impossibility of using shell elements in Abaqus/CEL, it was necessary to use solid continuum elements for the solid domain of the 3D case. Furthermore, only one layer of elements was allowed in the thickness of the leaflets, due to the fluid discretization requirements (as explained in the previous section). To verify the assumption on the number of elements in the thickness of the valve, two additional structural simulations were performed. Again, the geometry, element type, material and contact properties were consistent with the IB-FSI set-up. In the first case, a valve with one layer of elements in the thickness was realized, while in the second test two layers of elements were applied in the thickness. The first simulation resulted in a RVOT of 80ms (Fig 5, left panel), the second in a RVOT of 40ms. The value obtained for the two-layers-element thickness valve fell within the expected range of RVOT values (40-65ms) [8,9,29–31], while in the other case a longer RVOT was obtained. The use of one layer of elements in the thickness, therefore, introduced numerical stiffness in the valve, compared to the geometry with two layers of elements across the thickness. This tests confirmed that the use of one layer of elements in the thickness of the valve introduced a portion of delay in the valve kinematics. However, the global delay measured in the IB-FSI simulation was more significant than the delay due to the choice of the elements. Therefore, the element choice could not fully explain the origin of the significant time delay in the IB-FSI. To isolate the origin of the time delay, the effect of the fluid-structure interaction contact algorithm was investigated and the results are reported in S1 Appendix. Tests showed that the presence of the FSI algorithm in the tested release of Abaqus/CEL 12.0 introduced a time delay in the flow velocity curve comparable to the delay obtained in the IB-FSI case.

## Conclusions

In this paper we performed a critical comparison between two FSI simulation techniques for a heart valve with flexible leaflets. Several papers are available in the literature reporting results for the IB-FSI simulations, proving that the technique is suitable (within its intrinsic limitations) to model fluid-structure interaction scenarios where large displacements are involved [5–11, 13, 14, 17]. Over the past few years, particular attention has been focused on improving the computational technique, to obtain a more robust and reliable formulation [6, 12, 13]. To the best of our knowledge, this is the first study systematically comparing the performance of ALE and IB methods for solving the FSI problem of an aortic valve and reporting in detail the pros and cons of both methods for this specific case.

In our experience, due to the deformation of the fluid mesh in the ALE formulation in case of large displacements, the simulation of a heart valve with a fluid-structure approach seems to be infeasible using the selected mesh motion techniques. The IB-FSI provided a solution to the problem for the opening phase, and offers a significant advantage in terms of computational cost, even though the number of elements is much higher in the 3D IB-FSI, compared to the corresponding ALE-FSI. We nonetheless observed a significant time delay in the leaflets motion with the presented IB-FSI simulations and a “loss of void” in the vicinity of the leaflets. In depth analysis led us to conclude that these limitations are related to the solver used (in its particular release version), rather than being intrinsically due to the immersed boundary technique. As such, caution is warranted in extrapolating our observations and conclusions to other numerical solvers and/or software versions and other fluid-structure interaction problems.

## Supporting Information

S1 Appendix Compressibility effect on the IB-FSI analysis of heart valves. (DOCX)
