# Supplementary material for: Fluid-Structure Interaction Simulation of Prosthetic Aortic Valves: Comparison between Immersed Boundary and Arbitrary Lagrangian-Eulerian Techniques for the Mesh Representation
Source: PLoS One. 2016 Apr 29;11(4):e0154517. doi: 10.1371/journal.pone.0154517 (PMC4851392; doi:10.1371/journal.pone.0154517)
Supplement: S1 Appendix — (DOCX) [file pone.0154517.s001.docx]

**S1 Appendix: Compressibility effect on the IB-FSI analysis of heart valves.**

The stability of an explicit solver such as Abaqus/CEL for fluid-structure interaction simulations can be improved and enhanced by artificially introducing a limited amount of compressibility in the simulation, even though the blood is assumed to be an incompressible fluid [9, 14, 36, 37]. The compressibility of a fluid such as blood can be introduced by modifying the bulk modulus K_b_ of the blood or the speed of sound in the fluid c_f_. The two quantities are related via the density of the blood ρ by the equation:

$c_{f}=\sqrt{\frac{K_{b}}{\rho}}$ (eq. S1)

The estimated physiological bulk modulus is between 1 and 2.6 GPa [37], resulting in a c_f_ of 1570 m/s, assuming a K_b_ = 2.6GPa and the physiological density of the blood ρ = 1060 kg/m^3^. In previously documented literature works reporting on FSI simulations of cardiac valves the value of K_b_ had been lowered to 1% of its physiological value [9, 10, 14, 36, 37], to reduce the computational time and increase the stability of the simulation. This value of K_b_ results in a speed of sound in the blood of 157 m/s. In the following, we will refer to the compressibility introduced in our simulations by means of speed of sound in the blood, keeping the physiological value (c_f_ = 1570 m/s) and the value reported in literature (c_f_ = 157 m/s) as references.

As discussed in the paper, the 2D simulations were performed with a c_f_ = 157 m/s, a value previously used in literature. The need of a lower value for the speed of sound (c_f_ = 15.7 m/s) arose from the 3D test case, as it was not possible to perform the simulation with the higher value. This choice was verified with several tests, to ensure that the low c_f_ was not affecting the reliability of the overall FSI simulation.

The influence of the compressibility was evaluated with several tests, to ensure it did not deteriorate the quality and reliability of the results. As no reports on a similar case were found for the solver Abaqus/CEL, further investigations on a simplified 2D case are reported, to ensure that, at least in the cases where the analytical solution is available, the solver provides reliable results.

*Simplified 2D case*

As the applied reduction (one or two orders of magnitude) in the speed of sound did not influence the results of the 2D case (Fig 3d), the origin of the difference in the valve kinematics between the presented IB-FSI case, the ALE-FSI case and the expected values obtained from literature was not completely clear. Therefore, further investigations on a simplified geometry were performed, to isolate the influence of each term of the Navier-Stokes equations and relate each term with the influence of the compressibility factor. A 2D rigid and straight tube was used for the test cases, to be able to compare the results with the analytical solution. The dimensions and the location of the inlet and outlet surfaces were consistent with the 2D set-up described in the paper (length =130 mm, height = 20 mm). The boundary conditions were chosen depending on the type of test performed.

To study the effect of the inertial terms of the Navier-Stokes equations, the fluid was considered inviscid, a sinusoidal pressure curve at the inlet and a constant pressure at the outlet were imposed. By doing so, if the fluid is incompressible, the Navier-Stoke equation of the momentum can be reduced to:

$\frac{\partial v}{\partial t}=\frac{-\partial p}{\partial x}$ (eq. S2)

Where the velocity as a function of time $\frac{\partial v}{\partial t}$ depends only on the pressure distribution $\left( -\frac{\partial p}{\partial x} \right)$. More in detail, the imposed pressure curve (in blue) and the expected velocity curve (in red) are reported in figure (S1.a).

The parameters selected for the generation of the sinusoidal input were consistent with the simulated aortic region, with a period of 0.8s and a mean pressure of about 100 mmHg. This resulted in a peak velocity of about 1 m/s for the 2D case under analysis. The speed of sound in the blood used in the test cases were c_f_ = 157 m/s and c_f_ = 15.7 m/s, resulting in a Mach number of Ma = 3.6x10^-3^ and Ma=3.6x10^-2^, coherent with the Mach numbers obtained from the IB-FSI simulations performed in the 2D and 3D cases discussed in the paper.

The use of an initial step in Abaqus/CEL was fundamental to alleviate the numerical effects that the initialization of the curves have in the IB-FSI simulation. The results shown in the following were obtained after ramping the pressures of the IB simulation from a zero level to the mean pressure value, as also described in the paper (“Materials and Methods – Material properties and boundary conditions” section).

The analysis of the phase shift of the signals in the tested case provided additional insights on the simulated set-up (Fig S1, left panels). In the worst case scenario, with a mean pressure of 100 mmHg and a c_f_ = 15.7 m/s, the velocity curves at the inlet and outlet of the domain were coincident, as shown in figure S1.b. Therefore, the wavelength of the signal in this case was still much longer than the domain of the simulation. The comparison between the expected analytical solution and the curves obtained with different values of c_f_ is shown in figure S1.c (in red and in green). In this case, the phase shift between the analytical solution and the calculated solution was also negligible, with a maximum difference of 1.1% between the calculated curve and the analytical curve. By lowering the c_f_ even further, a mismatch in the amplitude of the curve was detected. In blue, the curve obtained with c_f_=8 m/s is reported, the Mach number in this case was about 0.09.

As the first ten harmonics of the fundamental frequency have an influence on the arterial waveform [S1], we tested the influence of the compressibility using the 5^th^, the 10^th^ and the 20^th^ harmonic as an inlet pressure profile. The simulations were performed with a c_f_=15.7m/s. The maximum error obtained for the 5^th^, the 10^th^ and the 20^th^ harmonic were respectively 5.9%, 9.4% and 19.4%. Despite the presence of 5.9% maximum error, the calculated 5^th^ harmonic had no significant phase shift between inlet and outlet, and between these and the analytical solution. When going to higher harmonics, the shape of the calculated waves changed compared to the expected curve. In figure S1.d-f, right panels the graphs of the inlet and outlet velocity of the 5^th^, 10^th^ and 20^th^ harmonic are reported. Note that the time scale differs for each graph.

**
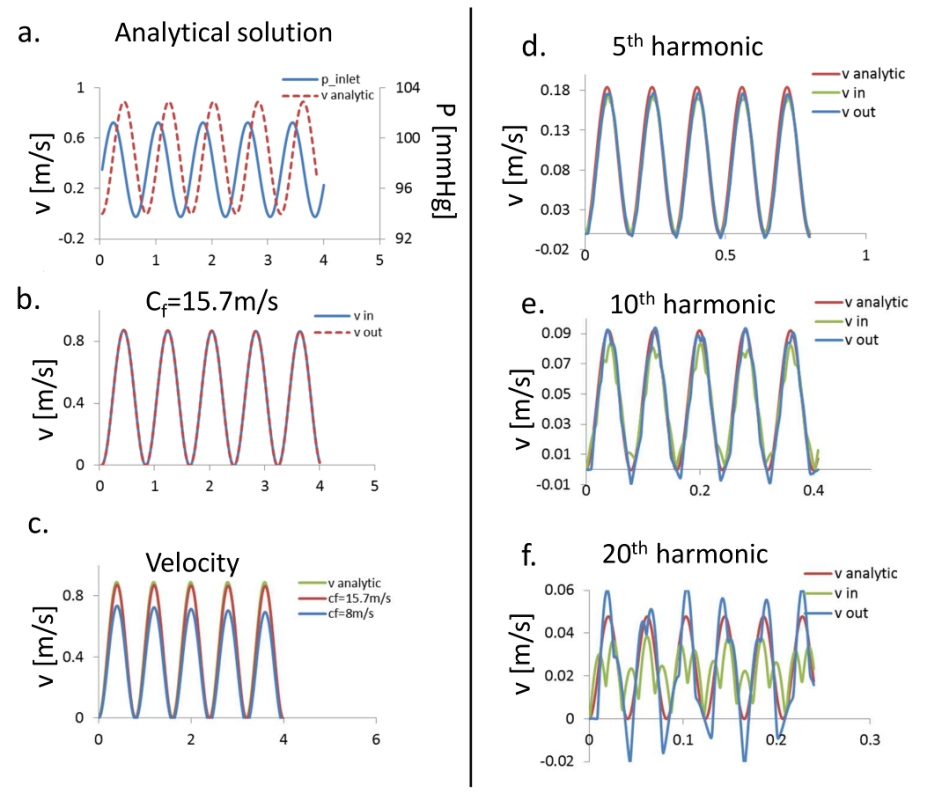
**

**S1 Figure:** left panel: (a) imposed pressure and analytically calculated solution. (b) Inlet and outlet velocities obtained for c_f_=15.7m/s. (c) Comparison between the analytical solution and the results of the simulations with low values of c_f_ (c_f_=15.7m/s and c_f_=8m/s). Right panel: results obtained imposing a sinusoidal pressure curve with the frequency of (d) the 5^th^, (e) the 10^th^ and (f) the 20^th^ harmonic respectively.

While the contribution of the 20^th^ harmonic on the cardiac pressure wave can be neglected, the 10^th^ harmonic could still have some influence on the results. In the worst case of cf = 15.7m/s, having an inlet signal with a frequency of f=12.5Hz led to a maximum error of almost 10% and a waveform of the calculated velocity slightly different from the expected curve.

In conclusion, for an inviscid fluid and for a pure sinusoidal inlet signal, the changes of the speed of sound in the blood did not affect the results for the range of values discussed in the paper (figure S1, left panels). However, using a more complex input signal such as a physiological pressure curve, some residual effects could be still present, due to the higher frequency harmonic components used in the simulations. This could be analyzed by imposing an inlet pressure with frequencies in the range of the harmonics of the real signal used in the calculation.

The second case performed was a steady state test, where the Poiseuille solution can be calculated. For a stationary laminar flow of an incompressible viscous fluid, the Navier-Stokes equations can be reduced to:

$\frac{\partial^{2}v_{x}}{\partial y^{2}}=\frac{\Delta P}{L\mu}$ (eq. S3)

Where v_x_ is the x-component of the velocity in the channel, ΔP is the pressure drop imposed, L the length of the channel and µ the viscosity of the fluid. A constant pressure difference of 1 mmHg was applied at the inlet/outlet of the geometry, to evaluate the effects of the viscous term in the equations. With this set of numerical experiments, we wanted to investigate separately the effects of the viscous term of the Navier-Stokes equations solved by Abaqus/CEL. Despite the presence of pressure fluctuations in the tube, the maximum velocity reached in the Abaqus/CEL set-up was 10% smaller than the maximum velocity obtained by Fluent, in a fully developed flow.

**Additional considerations over the time delay**

Having proven that the use of the compressibility was not the main cause of the time delay shown by the valve in the IB-FSI and having discussed the impact of the element type and number of element layers choice, further investigations were performed to identify the origin of the time delay. A possible source of error was the fluid-structure interaction algorithm used in the Abaqus/CEL release 12.0. The interaction between the two domains is enforced via the general contact algorithm, providing no-slip conditions to the fluid and a no-separation constraint for the two domains. To investigate this, a simple tube with rigid walls was simulated. The flow was chosen as viscous (µ=0.003Pa∙s), no-slip conditions were imposed at the walls and a sinusoidal inlet pressure curve was imposed, after ramping the pressure at the inlet and outlet to the mean pressure value of 100mmHg, consistent with the procedure used in the IB-FSI simulation of the valve. In the first set-up, the rigid walls were implemented by imposing a velocity constraint on the fluid, no real structural domain was included (therefore the term “no-walls” was used in the results to indicate this type of boundary conditions), resulting in a CFD simulation (fig. S2.a). In the second test-case the rigid walls were included in the simulation, the interaction between the fluid and the solid and the no-slip conditions were enforced with the contact algorithm (fig. S2.b).

The obtained velocity curves are reported in figure S2.c.


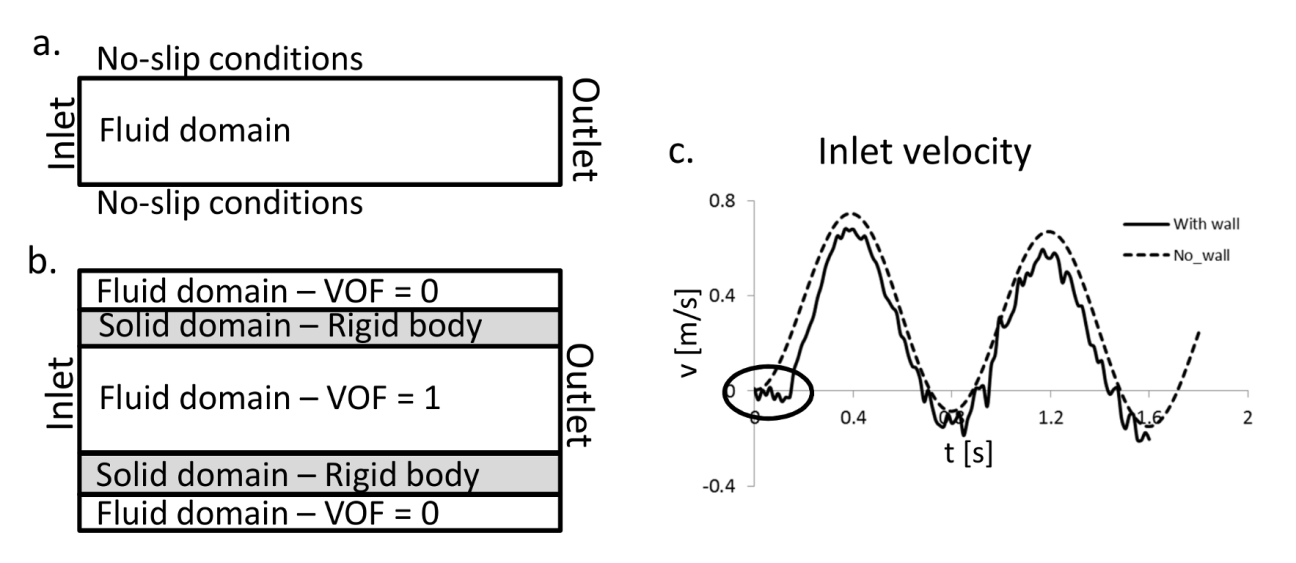


**S2 Figure:** (a) First set-up geometry. (b) Second set-up geometry. (c) Inlet velocity profile comparison: with and without walls. The delay is indicated with the circle.

The results of the simulation with no walls (dotted line) were in agreement with the expected solution. The presence of the fluid-structure interaction introduced some fluctuations in the velocity curve and, most important, a time-shift at the onset of the velocity curve, which then disappeared with the progression of the simulation. The initial delay of the FSI simulation in the test case was t=114ms, comparable to the time-delay obtained in the IB-FSI valve case. As in the IB-FSI simulation of the heart valve it was not possible to simulate multiple cardiac cycles (due to the discussed reasons), this effect could be still present in the results and therefore affect the kinematics of the valve.

**Conclusions**

The influence of the compressibility factor was tested in a 2D simplified Abaqus case, in both a steady state and a transient configuration. For the transient simulations, we can conclude that the reduction of the speed of sound did not significantly affect the results of the simulation, as the characteristic wavelength of the set-up was still much longer than the domain. The effects of the harmonics of the signal were also tested, to ensure that their influence was negligible. Furthermore, the Poiseuille flow test provided good results, with a 10% difference between the Fluent and the Abaqus solution. The viscous terms have a minor impact on the final fluid-dynamics of the set-up compared to the inertial terms. For a comparable velocity obtained in the Poiseuille test case and in the inertial test case, the pressure drop of the second scenario is 100 times greater than the first. Therefore, the contribution of the viscous terms for the 2D tube can be neglected when compared to the inertial terms.

Taking into account the results of the presented simplified test cases, the qualitative considerations over the Mach number and the results obtained from the 2D and 3D simulations, we can conclude that in the case of interest the compressibility did not compromise the overall reliability of the simulation. The inertial terms have been verified up to the 10^th^ harmonic frequency and the steady state simulation provided a solution within an acceptable error range. In conclusion, the time delay detected in the IB-FSI simulation of the heart valve could be related mainly to two factors: first, the choice of using one layer of elements in the thickness of the valve introduced numerical stiffness to the valve, which resulted in a slightly delayed kinematics. The second and main source of delay seemed to be related with the implementation of the contact algorithm of Abaqus/CEL (release 12.0) for the fluid-structure interaction, which introduced an initial time delay in the flow velocity. In the impossibility of simulating multiple cardiac cycles, the presence of an initial time delay affected the timing of the IB-FSI simulation, resulting in a longer RVOT for the aortic valve.

# References

# S1. Iaizzo P. Handbook of Cardiac Anatomy, Physiology, and Devices. 2^nd^ ed. Springer, 2009
